# A role of color vision in emmetropization in C57BL/6J mice

**DOI:** 10.1038/s41598-020-71806-0

**Published:** 2020-09-10

**Authors:** Jinglei Yang, Li Yang, Rongfang Chen, Yun Zhu, Siyao Wang, Xueqin Hou, Bei Wei, Qiongsi Wang, Yue Liu, Jia Qu, Xiangtian Zhou

**Affiliations:** 1 School of Ophthalmology and Optometry and Eye Hospital, Wenzhou Medical University, 270 Xueyuan Road, Wenzhou, 325027 Zhejiang China; 2 State Key Laboratory of Optometry, Ophthalmology and Vision Science, Wenzhou, Zhejiang China; 3 School of Optometry, Center for Eye Disease and Development, University of California-Berkeley, Berkeley, CA 94720 USA

**Keywords:** Colour vision, Retina

## Abstract

Spectral composition affects emmetropization in both humans and animal models. Because color vision interacts the effects of chromatic defocus, we developed a method to bypass the effects of longitudinal chromatic aberration by placing a spectral filter behind the optics of the eye, using genetic tools. Newborn C57BL/6J (B6) mice were reared in quasi-monochromatic red (585–660 nm) or blue (410–510 nm) light beginning before eye-opening. Refractive states and ocular dimensions were compared at 4, 6, 8, and 10 weeks with mice reared in normal white light. Cre recombinase-dependent *Ai9* reporter mice were crossed with *Chx10-Cre* to obtain *Chx10-Cre;Ai9* mice, expressing red fluorescent protein in retinal Cre-positive cells. *Ai9* offsprings, with and without Cre, were reared under a normal visual environment. Refraction and axial components were measured as described above. Expression levels of M and S opsin were quantified by western blotting at 10 weeks. Compared with those reared in white light, B6 mice reared in red light developed relative hyperopia, principally characterized by flattening of corneal curvature. Emmetropization was not affected by blue light, possibly because the reduction in vitreous chamber depth compensated for the increase in corneal curvature. Compared with *Cre*-negative littermates, the refraction and axial dimensions of *Chx10-Cre;Ai9* mice were not significantly different at the follow-up timepoints. M opsin levels were higher in *Chx10-Cre;Ai9* mice at 10 weeks while S opsin levels were not different. Red light induced a hyperopic shift in mouse refractive development. Emmetropization was not impacted in mice with perturbed color vision caused by intrinsic red-fluorescent protein, suggesting that color vision may not be necessary in mouse emmetropization when other mechanisms are present.

## Introduction

Myopia is the most common refractive abnormality in the world. The prevalence has increased significantly in recent decades as reported by numerous studies^1^. In 2000, the global prevalence was 22.9%, and it increased to 28.3% in 2010^2^. It is estimated that by 2050, the incidence of myopia worldwide will increase to 50% and that of high myopia (−5.00 Diopter [D] or more) to about 10%^2^. To explore ways to fight the global myopia epidemic, there is an urgent need to understand the etiology of this condition^3^.

To develop clear unaccommodated distance vision (emmetropia), the physical length of the eye must match the focal length so that images of distant objects fall on the retina. Mismatches between them cause the images to fall either in front of the retina, resulting in myopia; or behind it, resulting in hyperopia^4^. Low hyperopia is the most common type of refractive state at birth, not only in humans^5,6^ but also in some other animals such as chicks^7^, guinea pigs^5^, tree shrews^8^, and marmosets^9^. In a normal visual environment, the eye reaches a near-emmetropic refractive state that eliminates this early refractive error as the eye develops from neonate to adolescence. It is widely accepted that the process of emmetropization is active and dependent upon the visual experience. Further, growing evidence suggests that various ambient lighting conditions may affect emmetropization and ocular growth. Among these conditions, ambient luminance and spectral composition are major areas of research interest^4^.

The intensity of ambient lighting affects the endpoint of emmetropization. For instance, guinea pigs^10^ and chicks^11,12^ reared under high-intensity lighting (10,000 lux) develop a hyperopic shift of their refractive state. In contrast, chicks reared under low-intensity ambient lighting (50 lux) develop a myopic shift^12^. Additionally, bright light exposure prevents form-deprivation myopia in chicks^13,14^, rhesus monkeys^15^, guinea pigs^10^, and mice^16^. Although there is a lack of clear causality, similar findings have been reported in myopic children for whom higher myopic refractive errors were correlated with increased time in mesopic light^17^.

In addition, the ambient spectral composition also influences refractive development. Natural light is a mixture of lights with a wide range of wavelengths that form multiple chromatic images located at different distances from the retina. Specifically, for eyes emmetropized to mid-wavelength light (yellow–green, 550–570 nm), longer wavelengths are focused behind the retina, whereas lights with shorter wavelengths are focused in front of it. In chicks^18,19^, guinea pigs^20,21^, as well as in the invertebrate squid^22^, lights with longer wavelength tend to increase eye growth and induce myopic development while those with shorter wavelength induce slowed axial growth and hyperopic development. However in other studies, opposite results were found, i.e., that red light induced hyperopia in tree shrews^23^ and monkeys^24^.

Color vision is the ability to discriminate among colors based on the wavelength composition of the light. That ability is independent of light intensity and is mediated by cone photoreceptors in the retina. In human eyes, there are three types of cones, i.e., S, M, and L cones, which are sensitive to peak wavelengths of 440, 543, and 566 nm respectively with small variation depending on their exact opsin sequences. Previous experiments explored the role of chromatic information in refractive development, usually using environmental monochromatic or quasi-monochromatic lights in chickens^19,25–27^, guinea pigs^28,29^, tree shrews^23^, or rhesus monkeys^24,30^ (see review^31^). However, the effects of defocus and color information were mixed in these experimental designs. Thus changes in the eye growth could not be attributed solely to either the wavelength property or the color vision of the animals tested. Gawne et al. proposed that the eye responds to the differential focus of shorter and longer wavelengths in two fundamentally different ways^23^. One possibility is that the eye might use the focus of lights as a target, i.e., matching the focal plane of the dominant wavelength by growing longer when the ambient light is of longer wavelength while growing less when the dominant wavelength is shorter. The second possibility is that emmetropization could use the relativity of the chromatic arrangement of lights as a cue. In this case, if the long wavelengths are in better focus than the short wavelengths, the host animal could somehow identify the eye as myopic^23,30^ and decrease the growth rate of axial development to achieve emmetropia. Until now, it has not been clear if and how color vision is involved in emmetropization, i.e., if it is the focus or color of the ambient environmental light that controls the refractive development and eye growth.

The most commonly used myopia animal models, such as chicks, guinea pigs, and tree shrews, have greatly expanded our understanding of the mechanisms and controls of the refractive development. However, the lack of thorough genetic information in those species has limited the potential of the models in understanding the genetic and the epigenetic mechanisms of myopia development. In recent decades, the mouse has become a promising myopia model in which both genetic and environmental factors can be investigated. Thousands of genetically engineered lines and numerous molecular tools are now available. Despite poor visual acuity (estimated to be equivalent to 20/2000 human vision^32^), the mouse, which has only two types of retinal cones, M and S with peak sensitivity at 508 and 360 nm respectively^33^, is a relatively simple animal model to investigate the chromatic mechanism in emmetropization. In 2006, De la Cera et al. reported the optical aberrations in mouse eyes^34^, and we have investigated the development of the refractive status and ocular growth in C57BL/6J (B6) mice since 2008^35^. To date, there have been numerous applications of mice as a myopia model^36–38^.

In the present study, we first investigated the mixed effects of wavelength/focus and color vision on emmetropization in B6 mice by rearing them under quasi-monochromatic red and blue lights. We also introduced a novel mouse line, *Chx10-Cre;Ai9*, that expressed the red fluorescent protein tdTomato in the retina. That protein can be stimulated by visible light and form an intrinsic red filter, rendering objects seen by *Chx10-Cre;Ai9* mice as dark. Emmetropization of *Chx10-Cre;Ai9* mice was characterized to see if the process was influenced by isolated color vision. By comparing the results from the two experimental setups, we were able to further differentiate the roles of wavelength and color cues in mouse emmetropization.

## Materials and methods

### Mouse lines and husbandry

All animal studies were conducted in accordance with the Association for Research in Vision and Ophthalmology Statement for Use of Animals in Ophthalmic and Vision Research and approved by the Animal Care and Ethics Committee at Wenzhou Medical University, Wenzhou, China. B6 mice were obtained from Nanjing BioMedical Research Institute of Nanjing University. All mice were housed in temperature-controlled rooms with a 12/12-h light/dark cycle. *Chx10-Cre* (JAX Stock Number: 026200) and *Ai9* mice (JAX Stock Number: 007909) were obtained from the Jackson Laboratory (Bar Harbor, ME, USA). The *Ai9* mice were crossed with *Chx10-Cre* mice for multiple generations to generate a homozygous *Chx10-Cre;Ai9* mouse line. Genotyping was performed with complementary DNA isolated from the tails. The primers, reaction components, and cycling conditions were taken from the Jackson Laboratory website (www.jax.org).

### Experimental design

#### Experiment 1: refractive development in B6 mice under normal white or quasi-monochromatic lights

**Figure 1:**
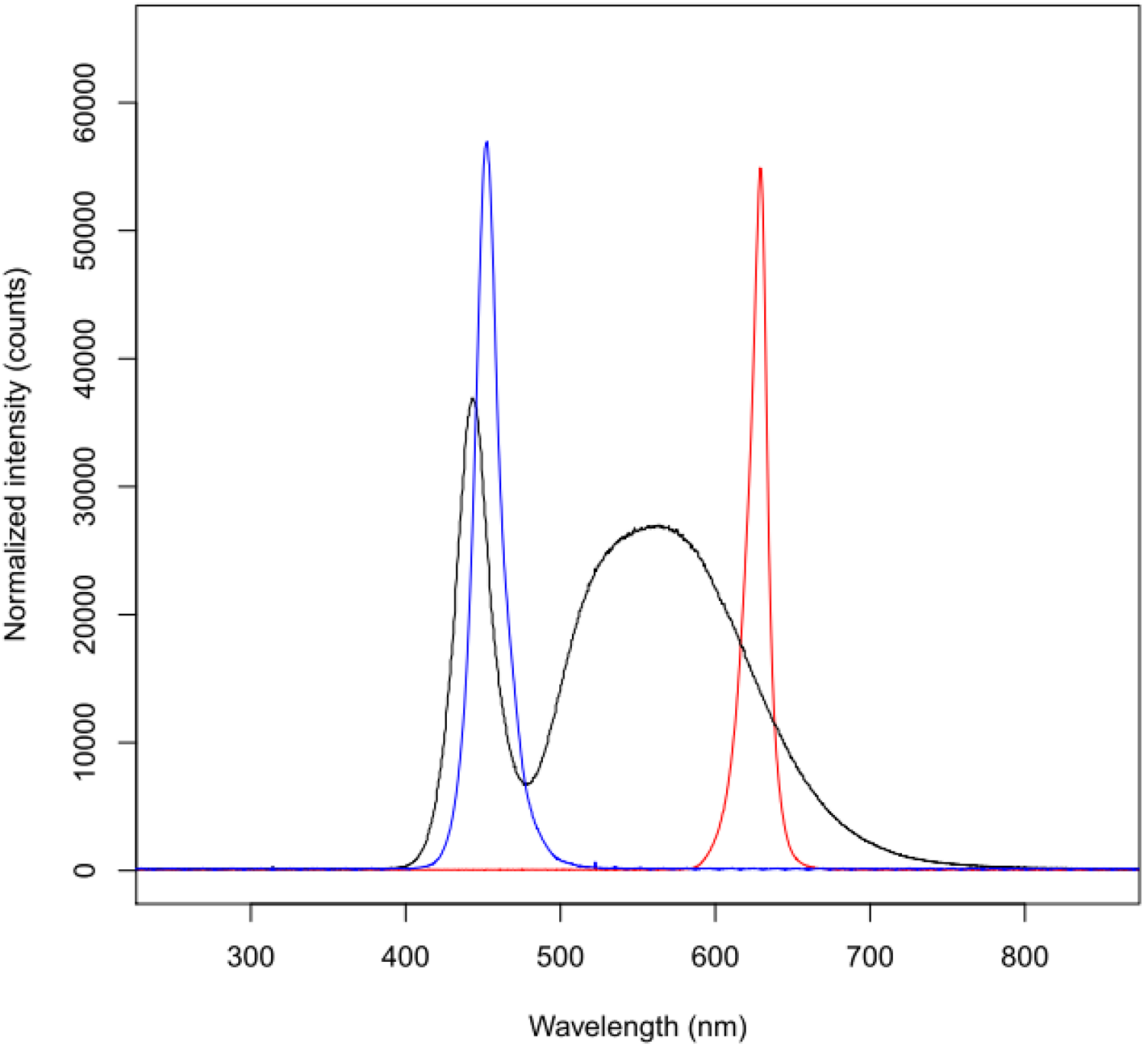
Spectral emission curves for the three types of LEDs used for mice rearing. The white light emitting LEDs had a broad emission spectrum (400–760 nm) with a high peak at 443 nm and a lower broad peak at 575 nm. The spectrum of the blue-emitting LEDs ranged from 410 to 510 nm with a sharp peak at 452 nm. The spectrum of the red-emitting LEDs spanned from 585 to 660 nm with a major peak at 629 nm.

Before eye opening at 14 days of age, B6 mice were randomly assigned to one of three visual environment groups: normal white light (*n* = 24), quasi-monochromatic red light (peak wavelength = 629 nm, *n* = 25), or quasi-monochromatic blue light (peak wavelength = 452 nm, *n* = 17). The mothers were kept in these cages for two or three days to enable the baby mice to nurse. Illumination was provided by light emitting diode (LED) tubes installed on the walls and ceiling of the cages to obtain a luminance of 275 ± 30 lux. Refraction and axial dimensions of all mice were measured every 2 weeks between 4 and 10 weeks of age. The emission characteristics of the LEDs were determined with a high-resolution spectrometer (HR 2000; Ocean Optics, Dunedin, FL, USA) (Fig. 1).

#### Experiment 2: refractive development of Chx10;Ai9 mice under white light

*Chx10-Cre;Ai9* mice (*n* = 17) and their *Cre-*negative littermates (*n* = 12) were raised in white light. As in Experiment 1, refraction and axial dimensions were measured every 2 weeks between 4 and 10 weeks of age. Appearance of the retinas with and without *Cre* was photographed by light microscopy.

### Electroretinogram (ERG) recording

We assessed the overall retinal function of B6 and Chx10-Cre;Ai9 mice to determine how they responded to red light. Scotopic and photopic flash ERGs were recorded with responses to white light stimuli as a control. ERGs were recorded with a custom-built Ganzfeld dome connected to a computer-based system (Q450SC UV; Roland Consult, Wiesbaden, Germany)^39^. The procedure was conducted as described previously^40^ with some minor modifications. All mice were reared under cyclic light/dark conditions and dark-adapted overnight before the experiments. General anesthesia was achieved with an intraperitoneal injection of ketamine (70 mg/kg) and xylazine (7 mg/kg), and the pupils were dilated with 1% tropicamide and 2.5% phenylephrine hydrochloride. A small amount of 2.5% methylcellulose gel was applied to the right eye, and a custom gold wire loop electrode was placed over the cornea as the active electrode. Needle reference and ground electrodes were inserted into the cheek and tail, respectively. Body temperature was maintained by placing the animals on a 37 °C warming pad during the experiment. ERGs to white light stimuli were recorded first to obtain control data. For scotopic flash ERGs, white light LED stimuli of five intensities (− 2.202, − 1.022, − 0.523, 0.477, and 0.977 log cd s/m^2^) were used without background lighting to generate and record rod-dominant responses and mixed rod- and cone-driven responses as the intensity increased. For each intensity between − 2.201 and − 0.523 log cd s/m^2^, ERGs were averaged from five single flashes. The interstimulus interval was 15 s. For intensities between 0.477 and 0.977 log cd s/m^2^, ERGs were averaged from three single flashes. The interstimulus interval was 40 s. Following 10 min of light adaptation with a rod-saturating background (1.398 log cd/m^2^), photopic stimuli were presented at 1 Hz, with − 2.201, − 1.022, − 0.523, 0.477, and 0.977 log cd s/m^2^ stimuli against the same background that the white lighting used for light adaptation. The responses to 50 flashes were averaged and filtered through a band-pass of 1–300 Hz. ERGs to red light stimuli (625 nm) were then recorded as above with some modifications. For scotopic flash ERGs, red light LED stimuli of three intensities (− 1.022, − 0.523, and 0.477 log cd s/m^2^) were used without background lighting. For photopic flash ERGs, the mice did not respond to red light stimuli with a background of white light.

Data acquisition and analysis were conducted according to our previous study^40^. Signals were band-pass filtered between 1 and 300 Hz for a- and b-waves. The amplitude of each ERG a-wave was measured from the baseline to the trough, and the implicit time was measured from the stimulus onset to the trough of the a-wave. The ERG a-wave amplitudes and implicit times were examined only at the three highest luminances. The amplitude of each b-wave was measured from the trough of the a-wave to the peak of the b-wave, and the implicit time was measured from the stimulus onset to the peak of the b-wave.

### Ocular biometric measurements

#### Refraction

Refraction was measured at the vertical pupil meridian in darkness using an eccentric infrared photorefractor designed by Schaeffel^41^. Briefly, each mouse was placed on a platform that was slowly adjusted until a clear first Purkinje image occurred in the center of the pupil, indicating an on-axis measurement. The data were then automatically recorded by the program and reported as the mean of at least three measurements.

#### Axial components of the eye

A custom-made spectral-domain optical coherence tomography (SD-OCT) system was used to measure the anterior chamber depth (ACD, from the posterior corneal surface to the anterior lens surface), lens thickness (LT, from the anterior lens surface to the posterior lens surface), vitreous chamber depth (VCD, from the posterior lens surface to the vitreous-retina interface), axial length (AL, from the anterior corneal surface to the vitreous retina interface), and the corneal radius of curvature of cornea (CRC). Each mouse was anesthetized with an intraperitoneal injection of ketamine hydrochloride (70 mg/kg body weight) and xylazine hydrochloride (10 mg/kg body weight) and then placed in a cylindrical holder for mounting on the positioning stage in front of a modified slit lamp. The SD-OCT scanning position was aligned along the optical axis of the eye with an X–Y cross-scanning system. The raw SD-OCT data were exported and analyzed using custom-designed software to obtain the axial components and CRC^42,43^. The mean of three repeated SD-OCT measurements for each eye was used for analysis.

### Immunostaining

*Chx10-Cre;Ai9* mice and their *Ai9 Cre-*negative littermates were sacrificed at four weeks of age. The eyes were enucleated and fixed for 30 min in cold phosphate-buffered saline (PBS) containing 4% paraformaldehyde and 3% sucrose adjusted to a pH of 7.4. Under a dissecting stereomicroscope, the anterior segment, including the cornea and lens, were quickly removed. The remaining eyecup was placed into PBS for another 30 min and then transferred into PBS containing 30% sucrose adjusted to a pH of 7.4 at room temperature for 30 min to allow the vitreous body replaced by an isosmotic sucrose solution. The entire eyecup preparation was then immersed for 30 min in room temperature PBS containing 30% sucrose, adjusted to a pH of 7.4. Afterwards, the eyecup preparation was embedded overnight in NEG50 (Thermo Fisher Scienti fi c, Waltham, MA). The fixed eyecup was cryo-sectioned into 10-µm thick slices that were collected on histo-bond slides and stored at − 40 °C before immunofluorescent staining. Prior to immunostaining, the cryosections were kept for 30 min at room temperature and then washed several times in 0.1 M PBS. These sections were coverslipped using antifade mounting medium (H1200, Vectorlabs) containing 4′,6-diamidino-2-phenylindole (DAPI) for staining of cell nuclei. Slides were viewed with a Zeiss LSM 780 confocal microscope.

### Western blot analysis

The western blot analysis was conducted as previously described^44^ but with some minor modifications. Retinas were lysed in 100 µl radio-immunoprecipitation assay buffer (Beyotime Biotechnology, Shanghai, China) with 1% phenylmethanesulfonyl fluoride (Beyotime Biotechnology) followed by mechanical and then ultrasonic homogenization. The mixture was centrifuged at 9600 g for 1 min and the supernatant was extracted. Equal volumes of protein extracts were subjected to 10% sodium dodecyl sulfate–polyacrylamide gel electrophoresis and transferred onto nitrocellulose membrane (Millipore, Billerica, MA). The membranes were blocked in 5% bovine serum albumin (Gibco, Thermo Fisher Scientific, Inc., Waltham, MA, USA) for 2 h at room temperature with agitation. Labeling of the S cone opsin, M cone opsin (rabbit anti-S opsin, dilution 1:1000, AB5407; rabbit anti-M opsin, dilution 1:1000, AB5405; respectively, EMD Millipore Corporation, Temecula, CA, USA), and α-tubulin (rabbit anti-α-tubulin, dilution 1:2000, ab52866, Abcam, Cambridge, MA, USA) with the primary antibodies were performed by incubation at 4 °C overnight. Membranes were then washed five times for 5 min each at room temperature in tris phosphate-buffered saline and incubated with secondary antibodies for 2 h at room temperature (IRDye 800CW goat anti-rabbit IgG, dilution 1:5000, 926-32211; Odyssey, Lincoln, NE, USA). Membranes were then washed as described earlier, followed by densitometric analysis of the protein bands by Image J (version 1.48 software; NIH, Bethesda, MD, USA). The values were normalized to corresponding α-tubulin loading controls.

### Statistical analysis

All parameters were expressed as means ± standard deviations. Only data collected from the right eyes were statistically analyzed. Intergroup differences of refraction and axial components were compared by ANOVA or the mixed linear model with missing data for repeated measurements^45^. Independent-samples t-tests were used to compare intergroup a- and b-wave amplitudes as well as the levels of S opsin and M opsin. All statistical analyses were performed with SPSS version 16.0 software (SPSS Inc., Chicago, IL, USA). *P* values less than 0.05 were considered to be statistically significant.

## Results

### Effect of quasi-monochromatic light on refractive development

Because mice were regarded as being less sensitive to red light^33^, we first used ERG recordings to determine if the B6 mice responded to red light (Supplementary Fig. 1). The mice had approximately half of the scotopic a- and b-wave amplitudes under red light stimuli than under white light stimuli (all *P* values < 0.05, repeated measures ANOVA). Under photopic conditions, the mice did not respond to red light stimuli with a background of white light.

#### Refraction

**Figure 2:**
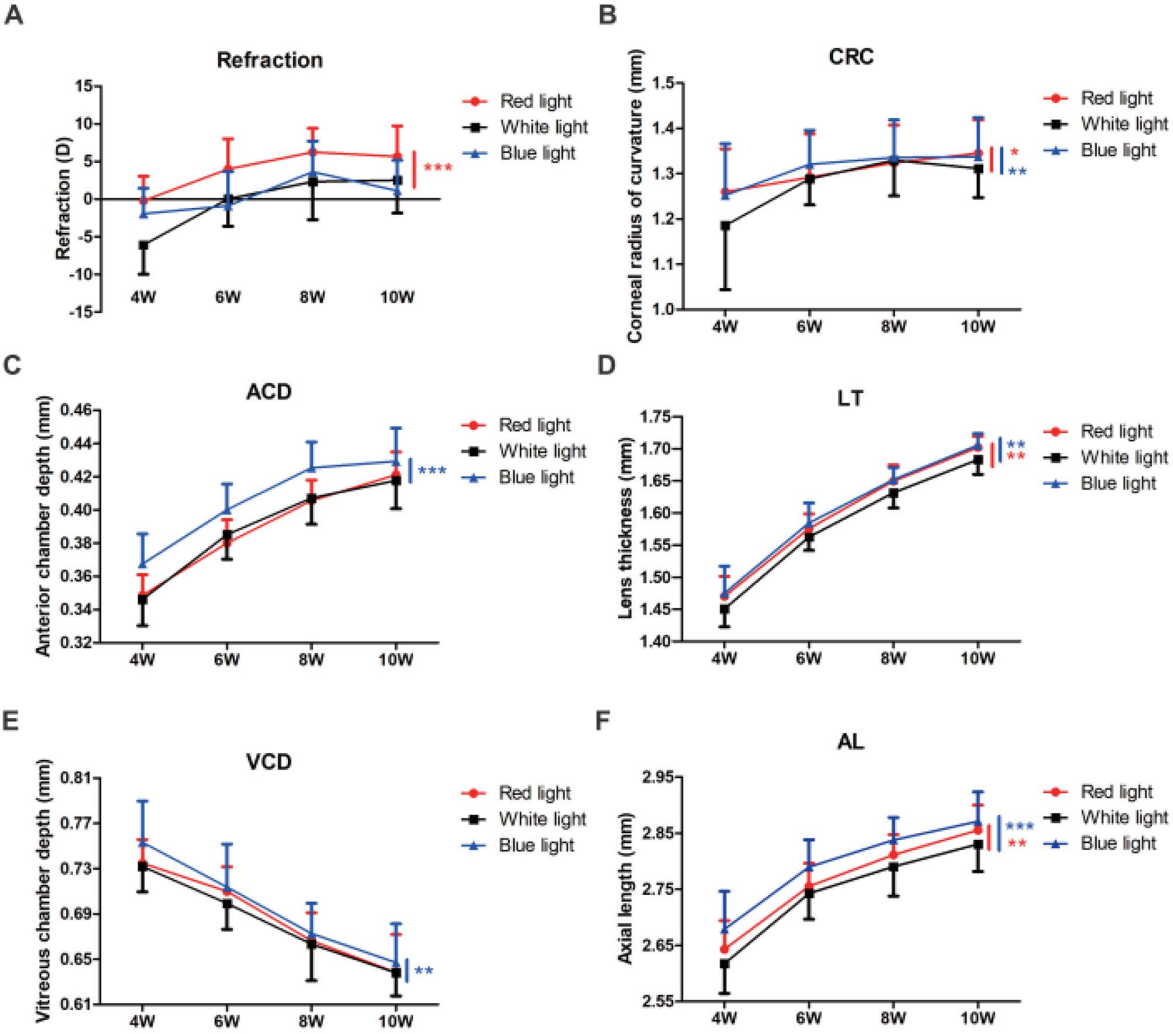
Effect of white, red, and blue light on refractive development and eye growth in B6 mice. (**A**) Mice reared in red light (*n* = 25) developed more hyperopia relative to those reared in white light. The pattern of refractive development with blue light (*n* = 17) was similar to that in white light (*n* = 24). The CRC (**B**), LT (**D**), and AL (**F**) in red and blue light increased more compared with those in white light. The increase in ACD (**C**) was greater and the decrease in VCD (**E**) was less in blue light compared with that in white light. Red and blue asterisks indicate statistical significance of differences between red or blue and white light. *P < 0.05; **P < 0.01: ***P < 0.001, mixed linear model. D, diopter; CRC, corneal radius of curvature; ACD, anterior chamber depth; LT, lens thickness; VCD, vitreous chamber depth; AL, axial length.

When raised in white light (Fig. 2A, Table 1), B6 mice were myopic (− 6.10 ± 3.90 D) at 4 weeks of age, followed by a hyperopic shift that became moderately hyperopic at 10 weeks (+ 2.53 ± 4.39 D). For mice reared in red light (Fig. 2A, Table 1), the refractions were − 0.21 ± 3.24 D and + 5.66 ± 4.07 D at 4 and 10 weeks respectively. The overall magnitude of the refractive change from 4 to 10 weeks for those reared in blue light (Fig. 2A, Table 1) was much smaller, (− 1.94 ± 3.38 D at 4 weeks and + 1.11 ± 4.05 D at 10 weeks). Mixed linear model analysis of these data showed that the hyperopic shift induced by red light was highly significant (*P* < 0.001) while the refractive change under blue light was not significant (*P* = 0.49).

**Table 1:** Ocular parameters of mice reared in white light and quasi-monochromatic red and blue light.

Group	*n*	Age (weeks)	Refraction (Diopter)	CRC (mm)	ACD (mm)	LT (mm)	VCD (mm)	ALcal (mm)	AL (mm)
White light	24	4	− 6.10 ± 3.90	1.19 ± 0.14	0.35 ± 0.02	1.45 ± 0.03	0.73 ± 0.02	2.53	2.62 ± 0.05
		6	0.04 ± 3.64	1.28 ± 0.06	0.39 ± 0.01	1.56 ± 0.02	0.70 ± 0.02	2.65	2.74 ± 0.05
		8	2.32 ± 5.06	1.33 ± 0.08	0.41 ± 0.02	1.63 ± 0.02	0.66 ± 0.03	2.70	2.79 ± 0.05
		10	2.53 ± 4.39	1.31 ± 0.06	0.42 ± 0.02	1.68 ± 0.02	0.64 ± 0.02	2.74	2.83 ± 0.05
Red light	25	4	− 0.21 ± 3.24	1.26 ± 0.10	0.35 ± 0.01	1.47 ± 0.03	0.73 ± 0.02	2.55	2.64 ± 0.05
		6	3.99 ± 4.01	1.29 ± 0.10	0.38 ± 0.01	1.58 ± 0.02	0.71 ± 0.02	2.57	2.76 ± 0.04
		8	6.23 ± 3.18	1.32 ± 0.08	0.41 ± 0.01	1.65 ± 0.03	0.67 ± 0.02	2.73	2.81 ± 0.04
		10	5.66 ± 4.07	1.35 ± 0.07	0.42 ± 0.01	1.70 ± 0.02	0.64 ± 0.03	2.76	2.86 ± 0.04
Blue light	17	4	− 1.94 ± 3.38	1.25 ± 0.11	0.37 ± 0.02	1.48 ± 0.04	0.75 ± 0.04	2.60	2.68 ± 0.07
		6	− 0.89 ± 4.56	1.32 ± 0.07	0.40 ± 0.02	1.58 ± 0.03	0.71 ± 0.04	2.69	2.79 ± 0.05
		8	3.62 ± 4.10	1.34 ± 0.08	0.43 ± 0.02	1.65 ± 0.02	0.67 ± 0.03	2.75	2.84 ± 0.04
		10	1.11 ± 4.05	1.34 ± 0.09	0.43 ± 0.02	1.71 ± 0.02	0.65 ± 0.03	2.79	2.87 ± 0.05

*n*, number of eyes; CRC, corneal radius of curvature; ACD, anterior chamber depth; LT, lens thickness; VCD, vitreous chamber depth; ALcal, calculated axial length, i.e., sum of ACD, LT, and VCD; AL, axial length.

#### CRC

The CRC of mice at 4 and 10 weeks were 1.19 ± 0.14 mm and 1.31 ± 0.06 mm in white light; 1.26 ± 0.10 mm and 1.35 ± 0.07 mm in red light; and 1.25 ± 0.11 mm and 1.34 ± 0.09 mm in blue light (Fig. 2B, Table 1). The overall corneal flattening induced by both the red and blue light environment were significant compared with the white light environment (*P* < 0.05, mixed linear model). The difference between the corneal flattening induced by the red and blue light environments was not significant (*P* > 0.05).

#### ACD

The ACD of mice at 4 and 10 weeks were 0.35 ± 0.02 mm and 0.42 ± 0.02 mm in white light; 0.35 ± 0.01 mm and 0.42 ± 0.01 mm in red light; and 0.37 ± 0.02 mm and 0.43 ± 0.02 mm in blue light (Fig. 2C, Table 1). While the ACD change induced by the blue light was significantly less compared to the white light (*P* < 0.001, mixed linear model), the change induced by the red light was not different from that with the white light (*P* > 0.05).

#### LT

The LT at 4 and 10 weeks were 1.45 ± 0.03 mm and 1.68 ± 0.02 mm in white light; 1.47 ± 0.03 mm and 1.70 ± 0.02 mm in red light; and 1.48 ± 0.04 mm and 1.71 ± 0.02 mm in blue light (Fig. 2D, Table 1). The lens thickening induced by both the red and the blue light were significantly more than with the white light (*P* < 0.01, mixed linear model), but not significantly different from each other (*P* = 0.567).

#### VCD

The VCD at 4 and 10 weeks were 0.73 ± 0.02 mm and 0.64 ± 0.02 mm in white light; 0.73 ± 0.02 mm and 0.64 ± 0.03 mm in red light; and 0.75 ± 0.04 mm and 0.65 ± 0.03 mm in blue light (Fig. 2E, Table 1). Thus, the decrease in VCD induced by blue light was significantly less than that induced by red light (*P* < 0.001, mixed linear model), while the effect from the red light was not different from that with white light (*P* > 0.05, mixed linear model).

#### AL

The AL of mice at 4 and 10 weeks were 2.62 ± 0.05 mm and 2.83 ± 0.05 mm in white light; 2.64 ± 0.05 mm and 2.86 ± 0.04 mm in red light; and 2.68 ± 0.07 mm and 2.87 ± 0.05 mm in blue light (Fig. 2F, Table 1). The overall induced axial elongation was significant for both the red and blue light environments compared with white light (*P* < 0.01, mixed linear model). Additionally, the difference between the axial change induced by the red and blue light was also significant (*P* < 0.01). Sum of ACD, LT and VCD was calculated as calculated AL, namely ALcal, which is consistent with the measured AL.

### tdTomato formed a red filter within the retina of *Chx10-Cre;Ai9* mice without changing retinal ERGs

**Figure 3:**
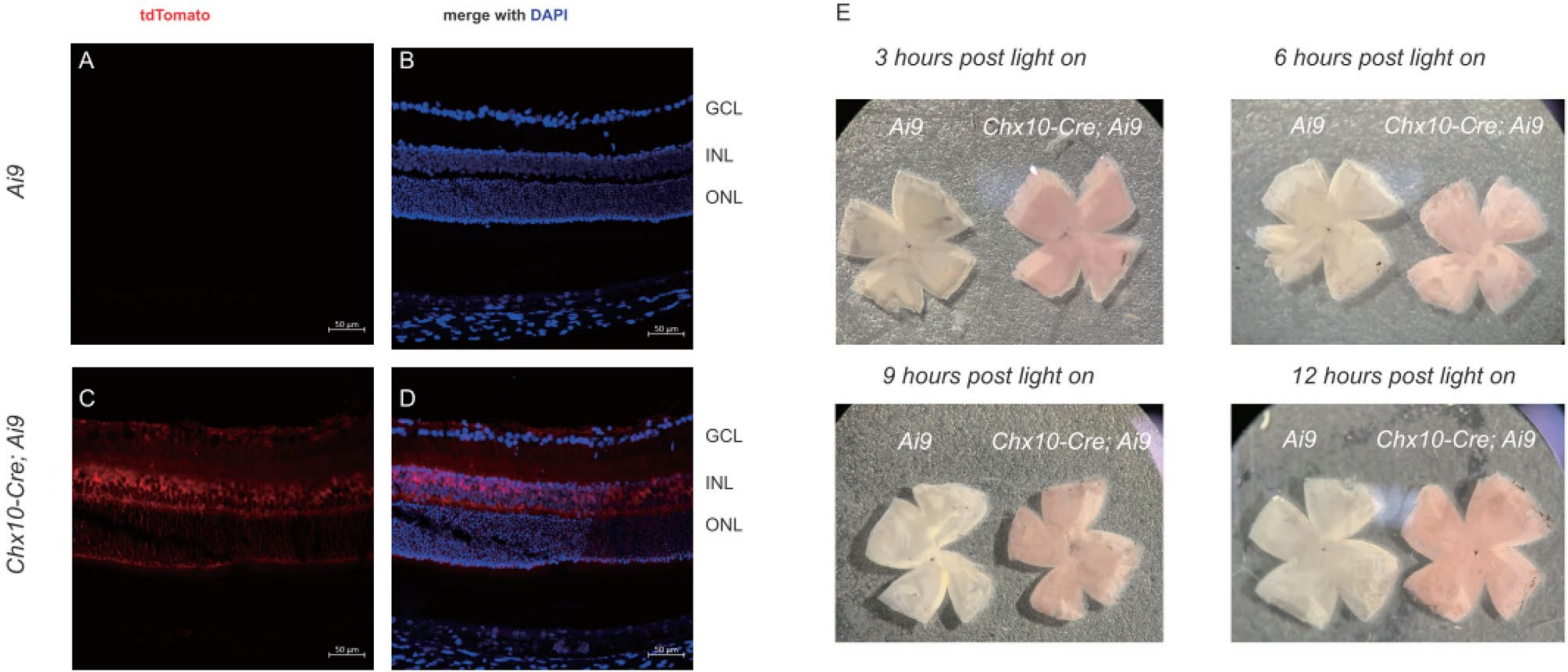
Fluorescent protein tdTomato formed a red filter within the retina in *Chx10-Cre; Ai9* mice. (**A**,**B**) In *Ai9* mice without Cre, the fluorescent tdTomato protein was not expressed in the retina. (**C**,**D**) In *Chx10-Cre;Ai9* mice, tdTomato was expressed in the retina after the STOP sequence was deleted by Cre. (**E**) The retinas from *Chx10-Cre;Ai9* mice appeared red after 3, 6, 9, and 12 h of exposure in room light under bright field microscopy. DAPI, 4′,6-diamidino-2-phenylindole. GCL, ganglion cell layer; INL, inner nuclear layer; ONL, outer nuclear layer.

*Ai9* is a reporter mouse line in which the expression of the tdTomato protein is dependent on Cre^46^. In *Ai9* mice, the placement of a STOP before the promoter silenced the expression of the tdTomato gene (Fig. 3A,B). In *Chx10-Cre;Ai9* mice, tdTomato was expressed in the outer nuclear layer, anterior to the photoreceptors (Fig. 3C,D). This layer of fluorescent protein was activated by light of 540 nm wavelength, which was included in room white light^47,48^. When observed under bright field microscopy, it provided the red hue of the retina after 3, 6, 9, and 12 h of room light exposure (Fig. 3E). Although the images were taken under light intensity of around 500 lux, which is higher than 275 lux (intensity of room where the mice were reared), it is reasonable to assume that this red layer acted as a filter that was positioned in front of the photoreceptors, and that the *Chx10-Cre;Ai9* mice saw all objects as dark.

**Figure 4:**
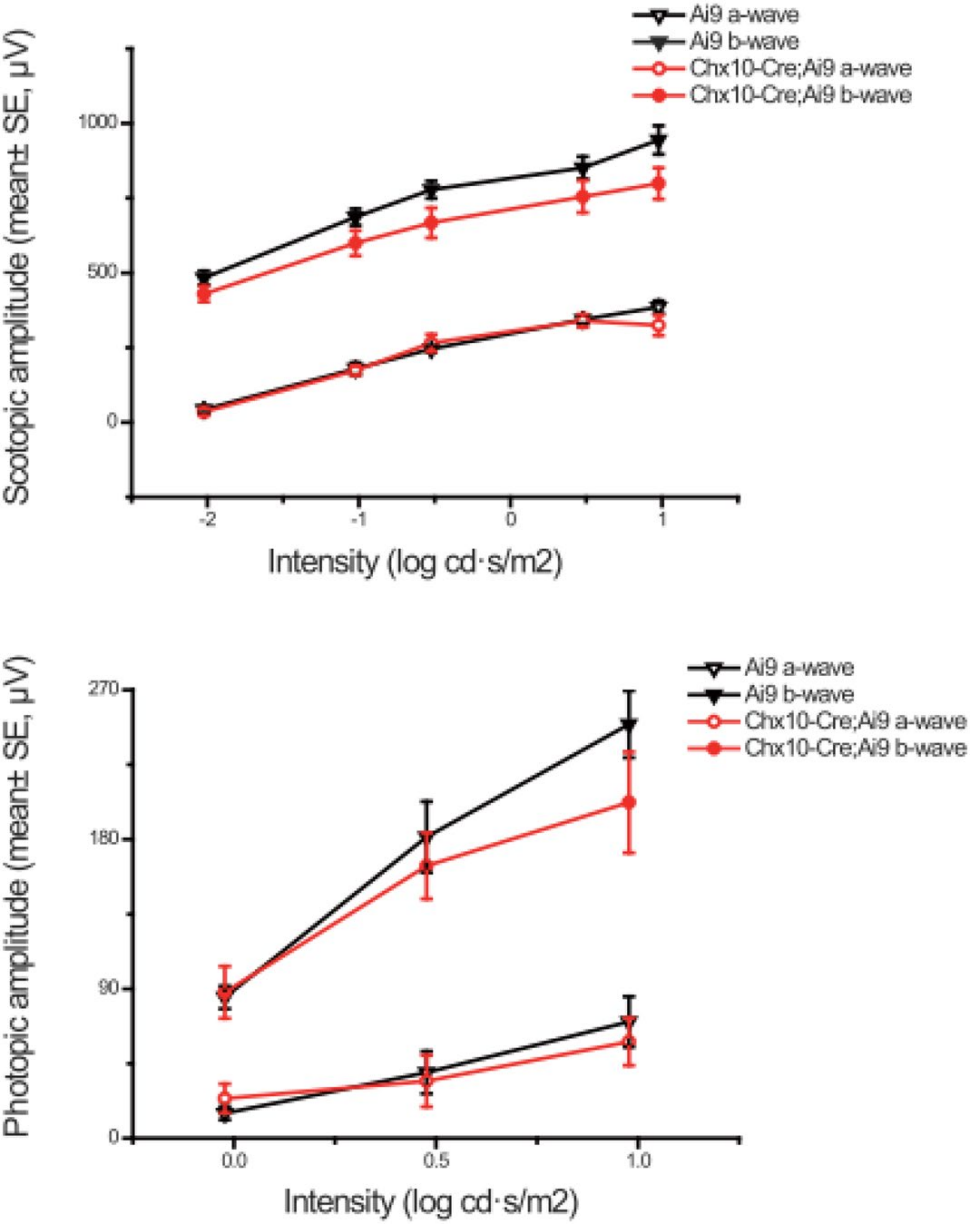
The tdTomato layer did not affect retinal function revealed by ERG recordings. There were no significant differences in a- and b-wave amplitudes for either scotopic or photopic conditions between Chx10-Cre;Ai9 and Cre-negative littermates (all *P* values > 0.05, repeated measures ANOVA).

To determine if this intrinsic red filter affected the retinal functions, ERGs were recorded and compared with those of *Cre*-negative *Ai9* littermates (Fig. 4). There were no significant differences in a- and b-wave amplitudes for either scotopic or photopic conditions between the Chx10-Cre;Ai9 and *Cre*-negative littermates (all *P* values > 0.05, repeated measures ANOVA).

### Emmetropization in *Chx10-Cre;Ai9* mice

**Figure 5:**
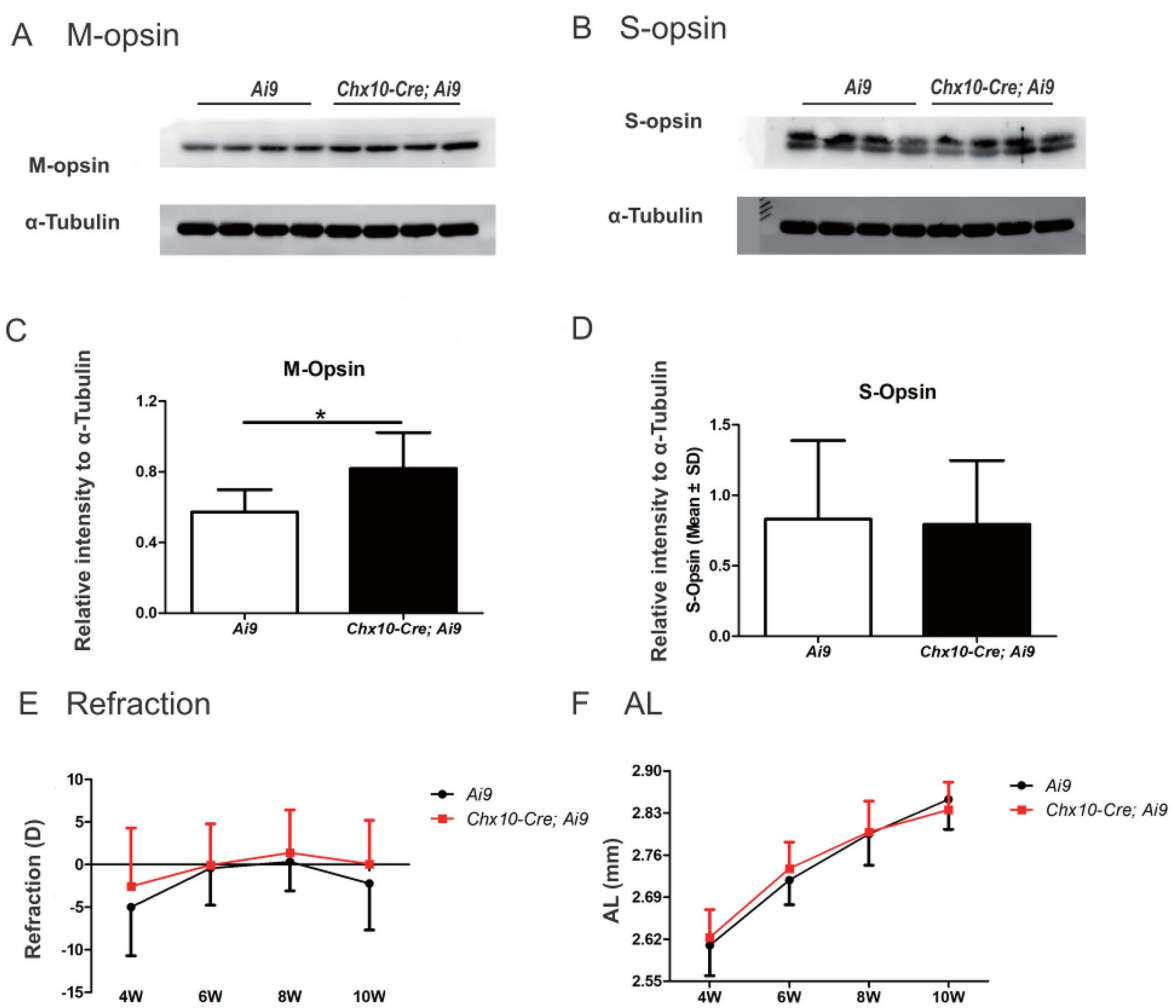
Opsin levels and refractive characteristics of *Ai9* and *Chx10-Cre;Ai9* mice. Retinal M (**A**,**B**) and S opsin (**C**,**D**) levels were quantified by western blotting relative to α-tubulin at 10 weeks of age (see original gels in supplementary file). Refraction (**E**) and axial length (**F**) of *Ai9* and *Chx10-Cre;Ai9* mice were recorded at 4, 6, 8, and 10 weeks. Only the M opsin level in the *Chx10-Cre;Ai9* mice at 10 weeks was higher than in the *Ai9* mice. Otherwise, emmetropization in *Chx10-Cre;Ai9* mice did not differ from the *Ai9* mice. *P < 0.05 by *t*-test.

The expression level of retinal M opsin, which is responsible for transduction of long wavelength light in mice^33^, was increased by 43.0% in *Chx10-Cre;Ai9* mice compared to *Ai9* littermates (*P* < 0.05, Fig. 5A,C). The expression level of S opsin, which transduces light with shorter wavelengths^33^, was not significantly different between the two strains (*P* = 0.887, Fig. 5B,D). At 4 weeks, the *Ai9* mice were myopic (− 5.0 ± 5.7 D, Fig. 5E). Some emmetropization occurred during the next 4 weeks, but at 10 weeks the eyes were still myopic (− 2.1 ± 5.5 D). The *Chx10-Cre;Ai9* littermates had a similar emmetropization process (Fig. 5E). The refraction at 4 weeks was − 2.6 ± 6.9 D, then it became hyperopic at 8 weeks before reverting to emmetropia at 10 weeks 0.1 ± 5.0 D. There were no significant differences in the refractive changes between the two strains (*P* = 0.362, mixed linear model). Similarly, there were no significant differences in AL between the two strains (*P* = 0.783, mixed linear model, Fig. 5F).

## Discussion

Numerous studies have investigated the effect of alteration in lighting parameters, like intensity, spectrum distribution, diurnal lighting rhythms, and spatiotemporal luminance modulation, etc., on emmetropization and eye growth. Whether or not the color vision of the animal is involved in this process has not been addressed because in previous experimental setups, wavelength information was always mixed with the detection and decoding of chromatic signals by the sensory retina. In the present study, we found that quasi-chromatic red light induced a hyperopic shift in emmetropization. By placing a spectral filter behind the optics of the eye, we developed a mouse strain that bypassed the effects of longitudinal chromatic aberration. On the basis of this unique anatomical arrangement, we propose that color vision is not necessary in the light-induced refractive shift that occurs during emmetropization.

In 1998, the Rodent Refinement Working Party suggested that mice were insensitive to red light^49^. However, numerous irradiance response curves to long wavelength light have shown that, while less sensitive than to short wavelength light, responses to bright, long wavelength can be detected by mice. Zhang et al. demonstrated that in mice white light induced sleep at a low intensity of 10 lux, while red light at the same intensity did not affect sleep–wake behavior^50^. However when the intensity was greater than 20 lux, red light exerted potent sleep-inducing effects. This result further confirmed that mice indeed detect long wavelength light if the intensity is high enough. In the present study, a significant hyperopic shift occurred in mice raised in quasi-monochromatic red light (620 nm) at 275 lux. This clearly demonstrated that the refractive development was affected by longer wavelength light. However it was unclear if the hyperopic shift of the refractive state, as well as the decelerated VCD growth that were noted as early as 4 weeks, were products of active readjustment of the endpoint or an altered response to unadjusted endpoint of emmetropization.

Despite a more significant hyperopic shift observed in B6 mice reared in red light, we did not find the expected opposite response in blue light. Compared to those under white light or blue light treatment, the hyperopic shift in B6 mice reared in red light was largely due to the significant flattening of the cornea. The slight AL elongation in red light was due to the thickening of the lens compared with mice raised in white light, and there were no significant differences in ACD and VCD between the white- and red-light raised mice. The decrease in VCD was smaller in the eyes exposed to blue light compared with that by white or red light. While the smaller decrease in VCD was statistically significant, the effect was not large enough to compensate for the changes in CRC and were not sufficiently physiologically significant to impact the refractive state. The hyperopic refraction stopped increasing in each group at 10 weeks mainly due to the compensating corneal flattening associated with the continuous increase of AL.

Based on the development of myopia in chicks^18,19^, guinea pigs^20,21^, and squid^22^, it was unexpected that the mice raised under red light showed a significant hyperopic rather than a myopic shift in refractive development. One possible explanation of the conflicting results is that the light intensity used in this study was relatively low compared to other studies. Because the sensitivity of mice to red light is much lower compared to white light, the brightness perceived by mice in red light was undoubtedly low. As low light intensity is generally considered to be a promotor for myopic development, the hyperopic development under low intensity red light in this study is unlikely to be explained by the luminance. Moreover, assuming that the retina targets the wavelength-specific focal plane to maximize the luminance contrast, the refraction induced by the longer wavelengths should bias eye development towards myopia. Thus it is unlikely that the wavelength may have played a dominant role in the observed refractive development. In addition, Rucker proposed that the effects of chromatic cues become more obvious when luminance contrast cues are degraded by astigmatic blur^51^. Therefore, we propose that the importance of color vision as a cue to emmetropization may be a more important at lower light intensities. The overall refractive change found in this study was also consistent with the results of an experiment in infant rhesus monkeys in which exposure to low intensity red light induced refractive excursion to hyperopia^30^. However it should be noted that different animal species possess drastically different capacities of corneal curvature change after birth. Consequently, the hyperopic shift seen with red light treatment as a result of corneal flattening in our mouse model may not be generalizable to other species.

In experimental myopia models, it is often difficult to study the role of the environmental lighting wavelength separately from the roles of decoding and color perception processes in animals. Thanks to transgenic mice, we were able to install a retinal red color filter in front of the photoreceptors. Doing so caused the red-transducing M opsin level to increase in *Chx10-Cre;Ai9* mice while the S opsin level did not change. The successfully induced red fluorescent protein in the retina theoretically would enhance red visual perception in *Chx10-Cre;Ai9* mice, in which the tdTomato protein acted as a filter and biased the color vision of mice without changing the focal planes of lights in various wavelengths. Interestingly, despite a significant hyperopic shift observed in B6 mice raised in red light, the emmetropization process of *Chx10-Cre;Ai9* was not significantly different from the *Ai9 Cre*-negative littermates. As a result, it is possible that the color vision may not be a necessary factor in refractive development when other mechanisms are present. Alternatively, the lack of a difference in refractive development between Ai9 and Chx10-Cre;Ai9 mice may follow from the fact that both have similar color vision, and that the Chx10-Cre;Ai9 mutant have re-adjusted their spectral sensitivity curve by more pigment and color adaptation.

In humans, color vision is mediated by three types of cones and two chromatic opponent mechanisms. Absence or alteration of any cone type is believed to cause color vision deficiency (CVD) and lead to dichromatic or anomalous trichromatic color vision. Qian et al. observed that among high school students, both protan and deutan subjects had a lower prevalence of myopia than those in the control group^52^. Another study by Rajavi et al. reported a significant correlation between lower visual acuity and CVD; however, there was no correlation between CVD and the type of amblyopia, refractive error, anisometropia, or strabismus among primary school children^53^. Ostadimoghaddam et al. also found that among school children, the prevalence of myopia and hyperopia were both lower in CVD subjects than that in control subjects^54^. The CVD patients suffered from disturbed color vision in normal white light^55^. Despite some inconsistency in the findings from clinical studies, these results suggest that at least color vision may not be essential in the process of emmetropization and the refractive development in humans.

The apparently contradictory results between the two phases of our study and that from other clinical data can be potentially explained by the differences in the light intensity utilized in the different experimental protocols. Most studies suggesting a non-essential role of color vision in refractive development were carried out under normal light intensity. In the present study, the mice were raised in 275 ± 30 lux, which was lower than the recommended light levels in laboratory animal rooms (350–400 lux at bench level)^56^. Based on the results of our two experiments, we hypothesize that the role of color cues is more important in refractive development under lower light levels and becomes less crucial when the light intensity reaches a normal level. This hypothesis could also explain some seemingly contradictory results in previous studies. For example, in the experiments by Smith et al. a long wavelength dominated visual environment was generated by having the monkey wear red spectacles that transmitted light above 650 nm^30^, while Liu et al. showed that monkeys exposed to red light had a slight tendency to develop myopia^24^. In the experiment by Smith et al., the red-light environment was achieved by the wearing of a filter that would have reduced the light intensity. In contrast, the red-light environment in Liu’s experiment was achieved under normal light intensity. The results of these two studies are consistent with our hypothesis that chromatic information as a cue for refractive development may be more efficiently utilized under low light intensity. Under normal light intensity, defocus and image degradation become more dominant cues for emmetropization.

While two-thirds of the light is absorbed by traditional physical color filters^57^, one limitation of the current study is that we were unable to determine how much light was absorbed by the red fluorescent protein layer before reaching the photoreceptors. Further, for the portion of light not absorbed by the red protein layer, it was unclear if it induced any physiological effects. Another limitation is that we did not investigate the role in ocular development of the red and blue wavelengths under light intensity gradients around the normal level. This will be explored in future studies to test the hypothesis that the importance of color cues diminishes under high luminance.

In summary, we found that, as in infant monkeys and tree shrews, long-wavelength light induced a hyperopic shift in mice under low light intensity. This result supports the idea that emmetropization does not necessarily target only the focal plane that maximizes luminance contrast. Color cues, which are considered non-essential in refractive development under normal light, may play a dominant role at lower light intensity.

## Supplementary information

Supplementary information.
