# Author Correction: A role of color vision in emmetropization in C57BL/6J mice

**DOI:** 10.1038/s41598-021-88185-9

**Published:** 2021-04-14

**Authors:** Jinglei Yang, Li Yang, Rongfang Chen, Yun Zhu, Siyao Wang, Xueqin Hou, Bei Wei, Qiongsi Wang, Yue Liu, Jia Qu, Xiangtian Zhou

**Affiliations:** 1 School of Ophthalmology and Optometry and Eye Hospital, Wenzhou Medical University, 270 Xueyuan Road, Wenzhou, 325027 Zhejiang China; 2 State Key Laboratory of Optometry, Ophthalmology and Vision Science, Wenzhou, Zhejiang China; 3 School of Optometry, Center for Eye Disease and Development, University of California-Berkeley, Berkeley, CA 94720 USA

Correction to: *Scientific Reports* 10.1038/s41598-020-71806-0, published online 10 September 2020

The original version of this Article contained an error, where the corresponding email of Jia Qu was incorrect. Correspondence and request for materials should be addressed to jia.qu@eye.ac.cn.

Furthermore, the third sentence of the Abstract contained an error, where the wavelength ranges for red and blue were reversed.

“Newborn C57BL/6J (B6) mice were reared in quasi-monochromatic red (410–510 nm) or blue (585–660 nm) light beginning before eye-opening.”

now reads:

“Newborn C57BL/6J (B6) mice were reared in quasi-monochromatic red (585–660 nm) or blue (410–510 nm) light beginning before eye-opening.”

These errors have now been corrected in the PDF and HTML versions of the Article.

