# Supplementary material for: A role of color vision in emmetropization in C57BL/6J mice
Source: Sci Rep. 2020 Sep 10;10:14895. doi: 10.1038/s41598-020-71806-0 (PMC7483713; doi:10.1038/s41598-020-71806-0)
Supplement: Supplementary file 1 — Supplementary information. [file 41598_2020_71806_MOESM1_ESM.docx]

**A role of color vision in emmetropization in C57BL/6J mice**

Jinglei Yang,^1,2*^ Li Yang,^1,2*^ Rongfang Chen,^1,2^ Yun Zhu,^1,2^ Siyao Wang^1,2^, Xueqin Hou,^1,2^ Bei Wei,^1,2^ Qiongsi Wang,^1,2^ Yue Liu,^3^ Jia Qu,^1,2,#^ and Xiangtian Zhou^1,2,#^

1 School of Ophthalmology and Optometry and Eye Hospital, Wenzhou Medical University, Wenzhou, Zhejiang, China

2 State Key Laboratory of Optometry, Ophthalmology and Vision Science, Wenzhou, Zhejiang, China

3 School of Optometry, Center for Eye Disease & Development, University of California-Berkeley, Berkeley, CA 94720, USA

These authors (*) contributed equally to this work.

^#^Corresponding Authors:

Xiangtian Zhou, School of Ophthalmology and Optometry and Eye Hospital, Wenzhou Medical University, 270 Xueyuan Road, Wenzhou, Zhejiang, China 325027; zxt@mail.eye.ac.cn.

Jia Qu, School of Ophthalmology and Optometry and Eye Hospital, Wenzhou Medical University, 270 Xueyuan Road, Wenzhou, Zhejiang, China 325027; jqu@wz.zj.cn.

Table of Contents:

1. Supplementary Figure 1: ERGs of C57L/B6 mice to red light stimuli
2. Supplementary Figure 2: Original western blotting images
3. ERGs of C57L/B6 mice to red light stimuli


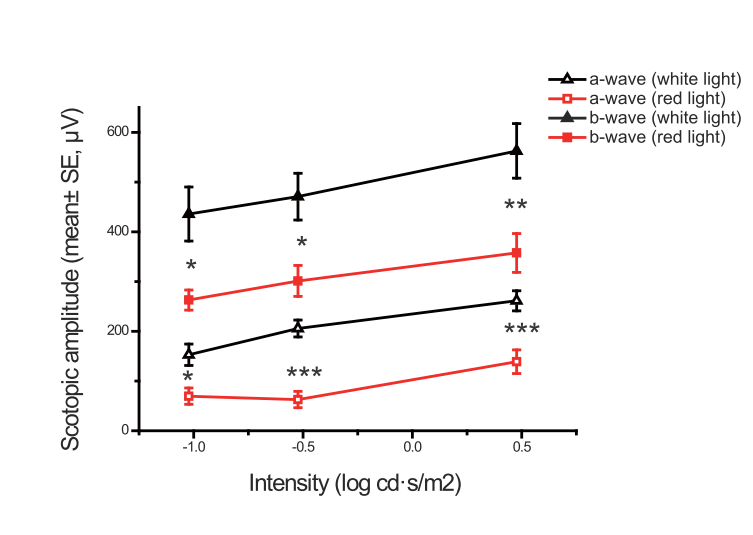


Supplementary Figure 1. In scotopic flash ERGs, C57L/B6 mice responded to red light stimuli with lower a- and b-wave amplitudes compared with white light stimuli. *: *P*<0.05; **: *P* <0.01; ***: *P* <0.001, independent-samples t-test.

1. Original western blotting images


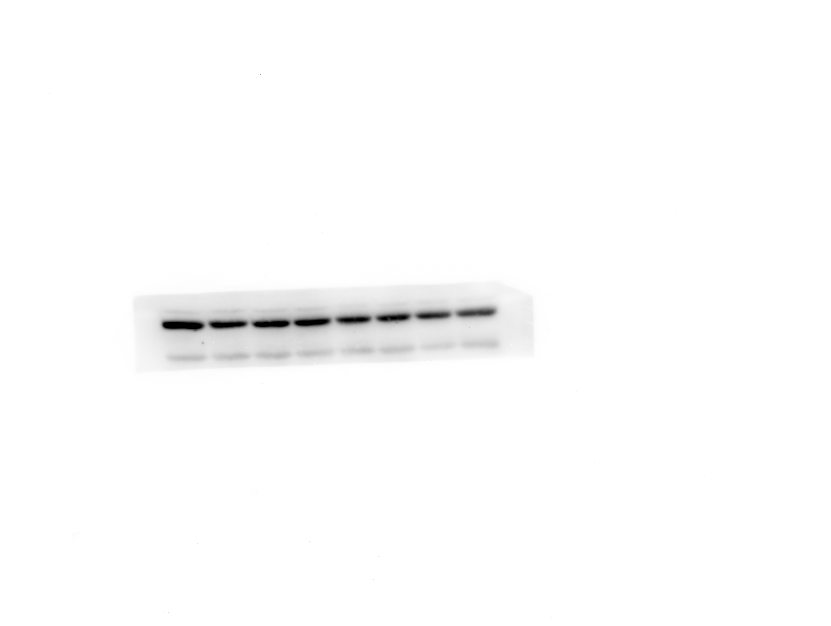


Supplementary Figure 2.1. M-opsin of *Chx10-Cre;Ai9* mice (lanes 1-4) and *Ai9* mice (lanes 5-8). The mirrored image was used in Figure 4.


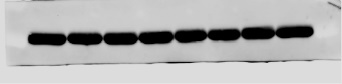


Supplementary Figure 2.2. α-Tubulin of *Chx10-Cre;Ai9* mice (lanes 1-4) and *Ai9* mice (lanes 5-8), used as internal standard for M-opsin. The mirrored image was used in Figure 4.


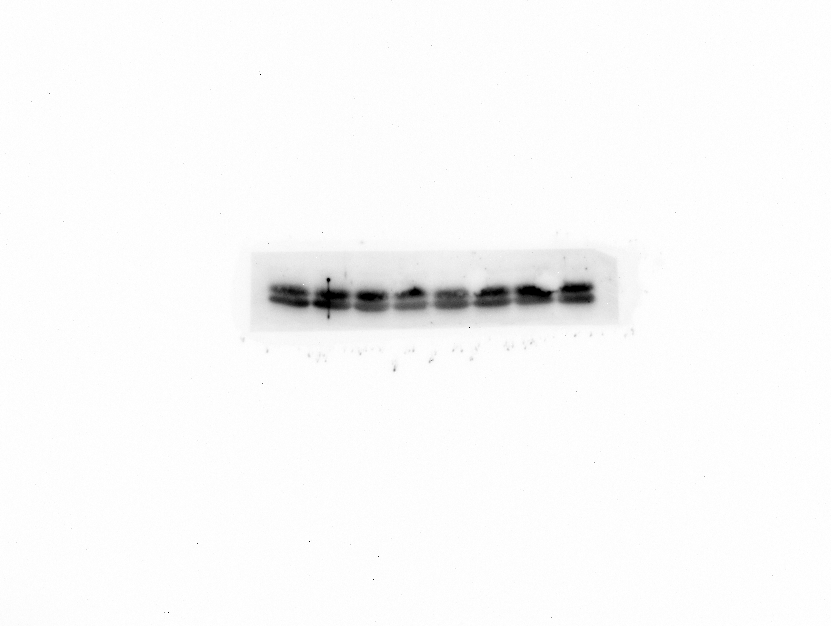


Supplementary Figure 2.3. S-opsin of *Chx10-Cre;Ai9* mice (lanes 1-4) and *Ai9* mice (lanes 5-8). The mirrored image was used in Figure 4.


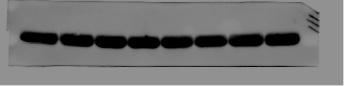


Supplementary Figure 2.4. α-Tubulin of *Chx10-Cre;Ai9 mice* (lanes 1-4) and *Ai9* mice (lanes 5-8), used as internal standard for M-opsin. The mirrored image was used in Figure 4.
